# Nanomolar clodronate induces adenosine accumulation in the perfused rat mesenteric bed and mesentery-derived endothelial cells

**DOI:** 10.3389/fphar.2022.1031223

**Published:** 2023-01-20

**Authors:** M. Verónica Donoso, Felipe Hernández, Rafael Barra, J. Pablo Huidobro-Toro

**Affiliations:** ^1^ Laboratorio de Farmacología, Departamento de Biología, Facultad de Química y Biología, Universidad de Santiago de Chile, Santiago, Chile; ^2^ Centro de Investigación Biomédica y Aplicada (CIBAP), Escuela de Medicina, Facultad de Ciencias Médicas, Universidad de Santiago de Chile, Santiago, Chile; ^3^ Centro de Nanociencia y Nanotecnología, Universidad de Santiago de Chile, Santiago, Chile

**Keywords:** clodronate, VNUT inhibitors, purinergic signaling, rat mesenteric bed, ATP/metabolite’s, adenosine deaminase

## Abstract

The vesicular nucleotide transporter (VNUT) is critical for sympathetic co-transmission and purinergic transmission maintenance. To examine this proposal, we assessed whether the bisphosphonate clodronate, claimed as a potent *in vitro* VNUT blocker, modified spontaneous and/or the electrically evoked overflow of ATP/metabolites and NA from mesentery sympathetic perivascular nerve terminals. Additionally, in primary endothelial cell cultures derived from this tissue, we also evaluated whether clodronate interfered with ATP/metabolite cell outflow and metabolism of N^6^-etheno adenosine 5′-triphosphate (eATP), N^6^-etheno adenosine (eADO), and adenosine deaminase enzyme activity. Rat mesenteries were perfused in the absence or presence of .01–1,000 nM clodronate, 1–1,000 nM Evans blue (EB), and 1–10 µM DIDS; tissue perfusates were collected to determine ATP/metabolites and NA before, during, and after perivascular electrical nerve terminal depolarization. An amount of 1–1,000 nM clodronate did not modify the time course of ATP or NA overflow elicited by nerve terminal depolarization, and only 10 nM clodronate significantly augmented perfusate adenosine. Electrical nerve terminal stimulation increased tissue perfusion pressure that was significantly reduced only by 10 nM clodronate [90.0 ± 18.6 (*n* = 8) to 35.0 ± 10.4 (*n* = 7), *p* = .0277]. As controls, EB, DIDS, or reserpine treatment reduced the overflow of ATP/metabolites and NA in a concentration-dependent manner elicited by nerve terminal depolarization. Moreover, mechanical stimulation of primary endothelial cell cultures from the rat mesentery added with 10 or 100 nM clodronate increased adenosine in the cell media. eATP was metabolized by endothelial cells to the same extent with and without 1–1,000 nM clodronate, suggesting the bisphosphonate did not interfere with nucleotide ectoenzyme metabolism. In contrast, extracellular eADO remained intact, indicating that this nucleoside is neither metabolized nor transported intracellularly. Furthermore, only 10 nM clodronate inhibited (15.5%) adenosine metabolism to inosine in endothelial cells as well as in a commercial crude adenosine deaminase enzyme preparation (12.7%), and both effects proved the significance (*p* < .05). Altogether, present data allow inferring that clodronate inhibits adenosine deaminase activity in isolated endothelial cells as in a crude extract preparation, a finding that may account for adenosine accumulation following clodronate mesentery perfusion.

## Introduction

The role of adenosine 5′-triphosphate (ATP) as an extracellular signaling molecule is firmly rooted. ATP metabolites such as adenosine 5′-diphosphate (ADP) or adenosine (ADO) are also extracellular messengers, the former mimicking ATP activity mainly through P2Y receptor activation, while the latter through its four ADO receptors. Some authors claim ADO is released as such to the synaptic cleft or is a consequence of ATP ectoATPase hydrolysis (Burnstock, 2018). In addition, purines also play a role as modulators of sympathetic transmission both in the brain and periphery *via* presynaptic mechanisms. From an evolutionary viewpoint, Estevez-Herrera et al. (2016) proposed that ATP is an ancient signaling molecule, functioning in cellular communications since early life stages. This thesis gets ample support from the Burnstock (2018) proposal recognizing the universal and primitive messenger role of ATP, as early as amino acids, and substantially earlier than amino acid-derived transmitters such as catechol, imidazole, or indol ethyl amines.

In support of the transmitter role of ATP, extracellular nucleotides have a short half-life in the synaptic space due to rapid hydrolysis to ADP, adenosine 5′-monophosphate (AMP), or ADO by several ectoATPases (Navarrete et al., 2014). Critical to the extracellular messenger ATP role, Moriyama and others described a vesicular nucleotide transporter (VNUT) as a key player in purinergic signaling (Sawada et al., 2008), a feature common to the recycling processes of most transmitters. The transporter ensures the maintenance of the purinergic synaptic transmission component including effects at peripheral neuroeffector junction sympathetic co-transmission. The VNUT is highly expressed in the brain and peripheral tissues such as the pancreas, liver, skeletal muscles, adrenals, and lungs (Moriyama et al., 2017). Although the crystal structure of the VNUT is lacking, it was early recognized to have a strong primary sequence homology to the brain glutamate transporter (VGLUT) as reported by Sawada et al. (2008). Based on indirect evidence, the first pharmacological characterization of the VNUT was based on VGLUT inhibitors such as Evans blue (EB) or 4,4′-diisothiocyanatostilbene-2,2′-disulfonate (DIDS).

The former is a symmetric azo dye with nanomolar potency, while the latter is a stilbene markedly less potent than EB requiring 100–300 times larger concentrations to block VNUT activity (Sawada et al., 2008). More recently, dichloromethylene diphosphonic acid (clodronate), a first-generation bisphosphonate, a drug clinically used for over 60 years to treat bone diseases, was alleged by Moriyama and Nomura (2018) to act as a selective and potent VNUT inhibitor. It was initially proposed that clodronate, based on its two negative phosphates, competes with chloride ions at an allosteric VNUT binding pocket with an affinity similar to acetoacetate, a prototype ketone body ligand (Moriyama and Nomura, 2018). Within few years, studies were extended to a series of novel bisphosphonates, including N-containing compounds, which also proved VNUT inhibitors in the Moriyama *in vitro* assay. Moreover, Kato et al. (2017) observed that clodronate impaired ATP vesicular release from neurons, astrocytes, or brain immune cells. Out of the several bisphosphonates investigated, clodronate proved the most potent with nanomolar potency as a VNUT blocker. Furthermore, Kato et al. (2017) highlighted clodronate’s analgesic potential with and a unique analgesic profile against neuropathic pain, a pharmacologic effect tentatively related to its alleged VNUT-blocking property. This finding allows relating purinergic signaling to chronic or neuropathic pain. Based on its pharmacological profile, clodronate may be considered a valuable research tool to explore ATP exocytosis in purinergic transmission, mimicking other drugs that block transmitter transport which turned critical to establish nerve endings recycling dynamics.

Therefore, whether clodronate interferes with the recycling of ATP in sympathetic nerve endings of the rat mesentery vascular bed was examined, where ATP is known to be co-released together with noradrenaline (NA) and constitutes an example of sympathetic co-transmission, Donoso et al. (2006) used this tissue to characterize sympathetic transmission in a peripheral neuroeffector junction and described its purinergic component. We proposed as a working hypothesis that clodronate, as a VNUT inhibitor, should decrease vesicular ATP storage in sympathetic nerve endings, reducing ATP overflow following electrical nerve terminal depolarization, impairing sympathetic co-transmitter overflow. Two aims guided the present investigation: 1) Assessing whether nanomolar clodronate rat mesentery perfusion reduced purine outflow induced by electrical depolarization of the perivascular nerve endings, decreasing ATP overflow to the mesentery perfusate. As a positive control EB, DIDS, and reserpine were used; the latter blocks the vesicular catecholamine transporter reducing monoamine storage in sympathetic neuroeffector junctions (Hillarp, 1960; Huidobro-Toro 1985; Mandela et al., 2010), 2) Examining whether clodronate increases ATP metabolism, an alternative explanation for the anticipated reduction in transmitter overflow to the mesentery perfusate. To this aim, we examined N^6^-etheno adenosine 5′-triphosphate (eATP) or N^6^-etheno adenosine (eADO) metabolism by primary cultures of endothelial cells derived from the rat mesentery since these etheno derivatives were previously characterized as exclusive endothelial cell ectoATPase substrates (Jackson et al., 2020). We extended the current study to assay adenosine deaminase (ADA) activity in cultured endothelial cells as in a calf intestinal mucosa type II crude powder.

The present results show that contrary to expectations, clodronate, even after a 3-h tissue incubation, did not reduce ATP or NA perfusate overflow elicited by electrical nerve terminal depolarization. Notwithstanding, the ADO output in mesentery perfusates was significantly increased. The increase in extracellular ADO might account for the reduction of the perfusion pressure elicited by the electrically evoked mesentery nerve terminal depolarization in preparations perfused with 10 nM clodronate. In contrast, EB, DIDS, and reserpine evidenced concentration-dependent reductions in the ATP/metabolites and NA outflow elicited by nerve terminal depolarization. Moreover, in primary cultures of endothelial cells or an ADA calf intestinal mucosa type II crude powder bioassay, 10 nM clodronate reduced ADA activity consonant with the significant ADO increase observed in mesentery perfusates.

## Experimental procedures

### Animals

Adult male Sprague–Dawley rats (250–350 g) bred at the Animal Reproduction Facility of the Faculty of Biological Sciences of the P. Catholic University were used, and the different protocols added 122 rats, distributed in the different group series. Animal handling conformed to animal care and NIH (United States) guidelines and procedures for laboratory animals’ use. The Faculty and the University ethical committees for use of animals for biological research approved the specific protocols designed and supervised our strict adherence to the subscribed guidelines, as certified by consent protocol number 121/2017-USACH. All efforts were enforced to minimize the number of rats used and their suffering. The animals were anesthetized with a mixture of ketamine: xylazine (25: 2.5 mg/kg) i.p.

### Drugs and chemicals

ATP and its metabolic degradation products as sodium salts as well as clodronate disodium, Evans blue (EB), 4,4′-diisothiocyanatostilbene-2,2′-disulfonate (DIDS), noradrenaline hydrochloride (NA), 3,4-dihydroxybenzylamine hydrobromide, reserpine chlorhydrate, quercetin, cyclodextrin adenine, and adenosine deaminase (ADA) from calf intestinal mucosa type II crude powder were purchased from Sigma-Aldrich Chemicals (Saint Louis, MO, United States). N^6^-etheno-purine standards for chromatographic column calibration or the development of specific experimental protocols (eATP and eADO) were previously synthetized in our laboratory. Chemicals used to prepare buffers and mobile chromatography phases were of analytical grade and purchased from Merck Chemicals (Darmstadt, Germany).

### Perfusion of the rat arterial mesenteric bed and collection of the mesenteric perfusate aliquots upon electrical depolarization of the perivascular nerve endings to quantify outflow of ATP/metabolites and NA

Upon animal anesthesia, the abdominal cavity was excised at the midline; the superior mesenteric artery was isolated and cannulated with plastic tubing to initiate Tyrode buffer perfusion at a 2 mL/min flow (37°C) bubbled with a mixture of 95% O_2_/5% CO_2_. The Tyrode solution composition was (mM): 118 NaCl, 5.4 KCl, 2.5 CaCl_2_, 1.2 KH_2_PO_4_, 1.2 MgSO_4_, 23.8 NaHCO_3_, and 11.1 D-glucose. With surgical scissors, the vascular mesenteric bed was disconnected from the intestines (McGregor, 1965) and transferred to a warmed tissue chamber to maintain Tyrode buffer perfusion; a pressure transducer was connected to the perfusion cannula. Continuous changes in perfusion pressure were monitored *via* a multichannel Grass polygraph (Donoso et al., 2006; Donoso et al., 2018b). In the meantime, the rats were killed under deep anesthesia by pneumothorax plus aortic bleeding (Donoso et al., 2006). The equilibration period consisted of a 40-min Tyrode buffer perfusion in the absence or presence of either clodronate, EB, or DIDS. Immediately thereafter, electrical stimulation of the perivascular nerve terminals, surrounding the superior mesenteric artery, commenced (20 Hz, 1 ms trains of 60 V) for 1 min; nerve depolarization was delivered *via* platinum electrodes connected to a Grass S44 stimulator. In all these protocols, the mesentery perfusate effluent was collected at 2-min intervals in pre-chilled 5-mL plastic tubes, 8 min before, during, and 22 min after the electrical depolarization. Sample collection commenced after the end of the equilibrium period. This protocol optimized perfusate collection to determine ATP/purines and NA overflow, before, during, and after the electrical nerve terminal stimulation. The co-transmitter overflow was quantified in the same perfusate aliquot.

For the clodronate protocol series, 30 separate mesenteries were perfused with .01–1,000 nM clodronate and eight separate tissues that were perfused without this drug. Likewise, for the EB series, 21 mesenteries were perfused in the presence of EB and eight mesenteries were perfused in its absence; likewise, for 1–10 µM DIDS perfusion series, 12 separate tissues were perfused with DIDS, while 10 were perfused without this chemical. For animals’ distribution to the different drug concentration group series, see Table 1; Supplementary Table S1. Additional experiments were conducted to extend the clodronate perfusion period for 180 min, four tissues were perfused with Tyrode buffer in the absence of clodronate, and four preparations were perfused in the presence of 10 nM clodronate. Fresh clodronate stock dilutions were prepared prior to each protocol. Non-drug-perfused mesenteries that served as controls were spaced along the course of the study period to rule out seasonal variations or other non-controlled variables that may cause data inconsistencies.

**TABLE 1:** Clodronate concentration-dependent effects on spontaneous and electrically evoked ATP/metabolites and NA overflow from the mesentery neuroeffector junction.

	Spontaneous overflow (pmol), X ± S.E.M.						
Clodronate nM							
	0 (n=8)	0.01 (n=5)	0.1 (n=5)	1 (n=5)	10 (n=7)	100 (n=4)	1000 (n=4)
ATP	8.05 ±2.49	16.64 ±8.55	52.97±20.45	4.31 ±1.69	38.98 ±18.41	10.67 ±3.69	20.23 ±10.81
ADP	15.53 ± 3.55	17.80 ± 5.19	55.75 ± 17.75	15.69 ± 7.52	33.92 ± 12.31	15.07 ± 1.88	27.31 ± 6.57
AMP	13.38 ± 4.74	3.54 ± 1.16	21.50 ± 4.77	5.53 ± 1.65	13.29 ± 3.23	14.54 ± 3.84	13.25 ± 3.62
ADO	7.12 ± 1.35	4.41 ± 0.82	15.82 ± 4.58	5.86 ± 0.89	8.84 ± 1.31	6.08± 1.45	13.40 ± 0.58 *
NA	0.65 ± 0.12	0.23 ± 0.13	0.58 ± 0.41	0.42 ± 0.37	0.55 ± 0.29	1.48 ± 0.60	1.51± 0.05 **

Total overflow elicited by nerve terminal depolarization (pmol), X ± S.E.M							
Clodronate nM							
	0 (n=8)	0.01 (n=5)	0.1 (n=5)	1 (n=5)	10 (n=7)	100 (n=4)	1000 (n=4)
ATP	91.59 ± 15.86	159.11 ± 71.72	244.70 ± 159.30	133.30 ± 40.33	141.89± 52.83	143.72 ± 21.22	778.38 ± 260.37
ADP	141.90 ± 35.58	148.20 ± 43.31	234.80 ± 152.10	232.83 ± 64.36	221.36 ± 39.74	326.06 ± 104.20	494.54 ± 168.78
AMP	197.20 ± 52.36	136.50 ± 36.08	170.20 ± 114.40	239.96 ± 38.57	280.01 ± 74.45	399.11 ± 51.35 *	278.95 ± 52.89
ADO	152.03 ± 26.86	98.73 ± 47.20	227.08 ± 94.46	186.45 ± 47.83	304.04 ± 35.88 **	481.12 ± 123.42	181.97 ± 35.64
NA	28.00 ± 5.68	24.06 ± 4.78	24.05 ± 2.28	23.46 ± 5.93	25.90 ± 3.86	19.98 ± 1.84	22.61 ± 4.32

In parenthesis, the number of preparations assessed. * *p* < .05; ** *p* < .01 unpaired t-test as compared to tissues perfused without clodronate.

### Reserpine treatment

To compare the effect of putative VNUT inhibitors, with an inhibitor of the vesicular NA transporter on the outflow of ATP/metabolites or NA, a series of protocols were performed using mesenteries from rats pretreated with either .2 or 2 mg/kg reserpine or vehicle. Matched groups of rats were treated with either vehicle (five rats) or a single sub-cutaneous dose of .2 mg/kg (three rats) or 2 mg/kg (five rats) reserpine; mesenteries from these rats were dissected 48 h after reserpine–vehicle treatment. Protocols were conducted as usual to examine spontaneous ATP/metabolites and NA outflow as well as electrically evoked co-transmitter outflow. The vehicle composition was as follows: acetic acid, propylene glycol, ethanol, and water, 2%, 4%, 4%, and 90% respectively.

### Quantification of the increase in tissue perfusion pressure elicited by perivascular nerve depolarization

In all series of mesenteries electrically depolarized, the increment in the mesentery pressure elicited by electrical depolarization was obtained by subtracting basal values, which normally were between 25 and 30 mm Hg; the values were expressed as the Δ increase in the mesentery perfusion pressure in mm Hg. This applied to tissues perfused with either drugs or only perfused with vehicle or Tyrode buffer.

#### Isolation and harvesting of primary endothelial cell cultures and protocol description

Additional five rats were used to harvest endothelial cells seeded in the 24 multi-well plates (Donoso et al., 2018a), and the protocol used to determine the spontaneous extracellular ATP/metabolites as well as following mechanical stimulation by cell medium displacement of the cell culture medium was described previously (Donoso et al., 2018a). To examine the effect of clodronate on extracellular ATP/metabolites, cell medium purines were analytically assessed in cell wells not exposed to clodronate, incubated with 400 µL Tyrode buffer 10 mM HEPES, or matched parallel wells incubated with Tyrode buffer 10 mM HEPES added with .01–1,000 nM clodronate for 40 min. This bisphosphonate concentration and its exposure time were chosen since in the mesentery perfusion protocols, it reduced the vasomotor response elicited by electrical nerve terminal depolarization. Some wells were not mechanically stimulated and represent spontaneous basal ATP/metabolite secretion, while other wells were mechanically stimulated by gently pipetting the cell media, a stimulus standardized previously that augments extracellular ATP in the cell media (Donoso et al., 2018a).

An aliquot (200 µL) of the cell media was retrieved for purine analysis from non-stimulated cell wells or 1 min after mechanical stimulation, a procedure we had used in the past to elicit ATP release to the cell media (Donoso et al., 2018a). At the end of the protocol, the wells were rinsed with Tyrode buffer 10 mM HEPES, and supernatants were discarded. The plate was placed at −20°C until Bradford’s protein determination was performed in each well. The extracellular ATP/metabolites in the cell media of each separate well were divided by the protein values of each well (pmol purines/mg protein), and expressed as percentage of the control (obtained in the absence of clodronate).

### Nucleotide metabolism by ectoATPases; protocols using etheno derivatives

To further examine endothelial cells’ nucleotide metabolism, we used eATP or eADO, which are exclusively metabolized by ectoATPases since these compounds do not freely cross cell membranes (Jackson et al., 2020). Four rats were used to harvest 24 multi-well plates with endothelial cell cultures, and the cells were added with 480 µL Tyrode buffer supplemented with 10 mM HEPES, in the absence or presence of 1–1,000 nM clodronate for 40 min. Two protocols were followed: in the first, endothelial cell wells were applied with 20 µL of 50 nM eATP, while in a second set of duplicate protocols, 20 µL of 25 nM eADO was applied. Stock solutions of both etheno derivatives were dissolved in HPLC water. The time course of the eATP/eADO cell nucleotide hydrolysis was within 30 min. The samples were retrieved at 1, 15, and 30 min, and the remaining eATP or eADO in the cell media were determined analytically by HPLC separation techniques followed by fluorescent detection as will be described. At the end of the protocol, the wells were rinsed with Tyrode buffer 10 mM HEPES, and the supernatants were discarded. The plate was kept at −20°C until Bradford’s protein determination assays were conducted. The eATP or eADO in the cell media of each separate well was expressed as pmol/mg protein. As control for these experiments, the samples of eATP or eADO were added to a parallel set of test tubes containing 480 µL of Tyrode buffer supplemented with 10 mM HEPES without cells; in this case, the results were expressed as pmol/mg protein, which corresponded to the average protein of the 24 multi-well plates with cell results that are expressed as the percentage of the respective eATP or the eADO concentrations.

### ADA activity

The colorimetric ADA enzyme assay described by Giusti and Galanti (1984) was followed. The procedure titrates the ammonia generated which can be determined by the indophenol reaction, synthesizing a blue-colored compound monitored spectrophotometrically. Two separate protocols assessed ADO metabolism: 1) ADO incubation with mesentery cells. Cell incubation was performed in sodium phosphate buffer 50 mM, pH 6.5, 37°C, in the absence or presence of 1–1,000 nM clodronate, adding exogenous ADO as a substrate. Three separate rats were used to harvest the 24-well plate cultured cells to achieve these protocols. The cells were incubated for 60 min with 10 mM ADO to start the enzyme reaction in excess substrate, and the chemical reaction was stopped in due time by retrieving a 400 µL aliquot of the cell media which was added to a test tube containing 1.4 mL phenol–sodium nitroprusside (106 mM phenol; .17 mM sodium nitroprusside). The samples were vortexed and 1.4 mL hypochlorite solution was added (11 mM of NaOCl, 125 mM NaOH). The samples are vortexed again and incubated for 30 min at 37°C prior to 620-nm spectrophotometric monitoring. A standard calibration curve was built with 3–300 µM ammonium sulfate in sodium phosphate buffer. At the end of the protocol, the wells were rinsed with sodium phosphate buffer, and the supernatants were discarded. The plate was stored at −20°C until Bradford’s protein determinations ensued, and the resulting cell medium ammonia of each separate well was divided by its protein content. The results of these studies are expressed as percentage of the ammonia produced by the reaction in the absence of clodronate, as compared to samples incubated with varying clodronate concentrations. As internal enzyme inhibition, 10 or 30 μM quercetin was used (Melzig, 1996). The vehicle for quercetin was β-cyclodextrin (18.5 mg/1 mL H_2_O). 2) crude enzyme assay. Reactions were performed in 96-well plates with the 6.25 mU/mL enzyme incubated in sodium phosphate buffer 50 mM, pH 6.5, 37^o^C, with reagents added in the following order: first, 10 μL of the several inhibitors used, second, 10 μL of the enzyme dilution (.78–50 mU/mL), and last, 10 μL of ADO (.01–20 mM). The reaction was incubated for 60 min at 37°C. The reaction was stopped by adding 93 µL phenol–sodium nitroprusside (106 mM phenol; .17 mM sodium nitroprusside), vortexed, and 93 µL hypochlorite solution was added (11 mM of NaOCl in 125 mM NaOH). The samples are vortexed again and incubated for additional 30 min at 37°C prior to 620-nm spectrophotometric readings. A standard calibration curve was built with 3–300 µM ammonium sulfate in sodium phosphate buffer. Test inhibitors used were 1–1,000 nM clodronate, 1 mM quercetin (El-Said et al., 2022), vehicle for quercetin β-cyclodextrin (18.5 mg/1 mL H2O), and .7 mM adenine (Alunni et al., 2008).

### Analytical procedures for ATP/metabolites and NA quantifications

#### Purine analytical determinations

Purines were quantified following a chemical reaction with chloro-acetaldehyde to generate the corresponding fluorescent N^6^-etheno purines. To this aim, a 200-µL aliquot of the 4-mL sample perfusate retrieved or the cell media from cell wells were used to react with 100 µL buffer phosphate–citrate, pH 4, and 10 µL of chloro-acetaldehyde. The samples were heated in a dry bath for 40 min at 80°C, a procedure that synthetized fluorescent derivatives which allowed picomolar detection of eATP, eADP, eAMP, and eADO. The chemical reaction was stopped by ice chilling for 5 min. The samples were stored at 4°C and analyzed within 24 h. HPLC procedures were used to separate and identify purines using the appropriate standards as previously detailed (Buvinic et al., 2007). The Merck-Hitachi HPLC instrument was used, equipped with a fluorescence detector at excitation and emission wavelengths of 233 nm and 415 nm, respectively. A 20 μL aliquot of each sample was injected into a Chromolith HPLC column equilibrated with the mobile phase at a flow of 1.5 mL/min (.2 M Na_2_HPO_4_, .2 M NaH_2_PO_4_, and 5 mM tetrabutylammonium; pH 6). A calibration curve was performed daily, and linearity was routinely attained between .0012 and 10 pmol/20 μL purines. The ATP/metabolite overflow to the mesentery perfusate was expressed as pmol collected per 4 mL fraction or was expressed as the total overflow accessible to the perfusate after electrical depolarization (fraction addition during and after perivascular nerve depolarization subtracting the spontaneous efflux); values were expressed as pmol.

#### NA determinations

Of the 4 mL of the mesentery perfusate collected, a 200-µL aliquot sample was separated on ice for the purine assay; the residual 3.8 mL was supplemented with 1.75 pmol 3,4-dihydroxybenzylamine, used as an internal standard. The luminally accessible NA was concentrated by Sep-Pak C-18 reverse-phase cartridges (Waters, Milford, MA, United States) eluted with the HPLC mobile phase used to assay NA by electrochemical detection. The eluates were evaporated to dryness and stored at −20°C. The samples were reconstituted in 200 µL water and quantified by HPLC, using the Merck L6200 A, Intelligent Pump, the Eicom electrochemical detector ECD-700S. A 100 μL aliquot of each sample was injected into a LiChroCART 125-4 HPLC column equilibrated with the mobile phase at a flow of 1.0 mL/min (.1 M NaH_2_PO_4_-H_2_O, .9 mM Triplex III, .66 mM 1-octanesulfonic acid, and acetonitrile 15 mL per liter; pH 2.8). A calibration curve was performed, and linearity was routinely attained between .35 and 5 pmol/100 μL NA and .5–7.0 pmol/100 μL 3,4-dihydroxybenzylamine. The released NA overflow was expressed as pmol collected per fraction (4 mL). The total overflow accessible to the perfusate following electrical nerve endings depolarization (amount obtained by adding all the fractions, after normalizing by subtracting the spontaneous NA efflux, values attained prior to the electrical depolarization), is expressed in pmol, as shown in Donoso et al. (2006).

### Statistical analysis

The results are expressed as the mean average values ± standard error of the mean. For statistical analysis, GraphPad Prism version 8.0.0 for Windows, GraphPad Software, San Diego, California United States, was routinely used. Normal data distribution was determined by the Shapiro–Wilk test. To compare two sets of data with normal distribution, the unpaired t-test or unpaired t-test with Welch´s correction was used in cases of variance differences. The Mann–Whitney test was used to compare two sets of data with non-parametric distributions. In the case of normal distribution, time course protocols used the one-way ANOVA, and in non-parametric distributions, the Kruskal–Wallis test was followed. The two-way ANOVA compared time course protocols of non-drug treated versus those treated with drug applications. In all cases, the significance level was set with an α value of less than .05.

## Results

### Changes in spontaneous or electrically evoked purines or NA overflow elicited in the presence of .01–1,000 nM clodronate: Time course studies

#### ATP

**FIGURE 1:**
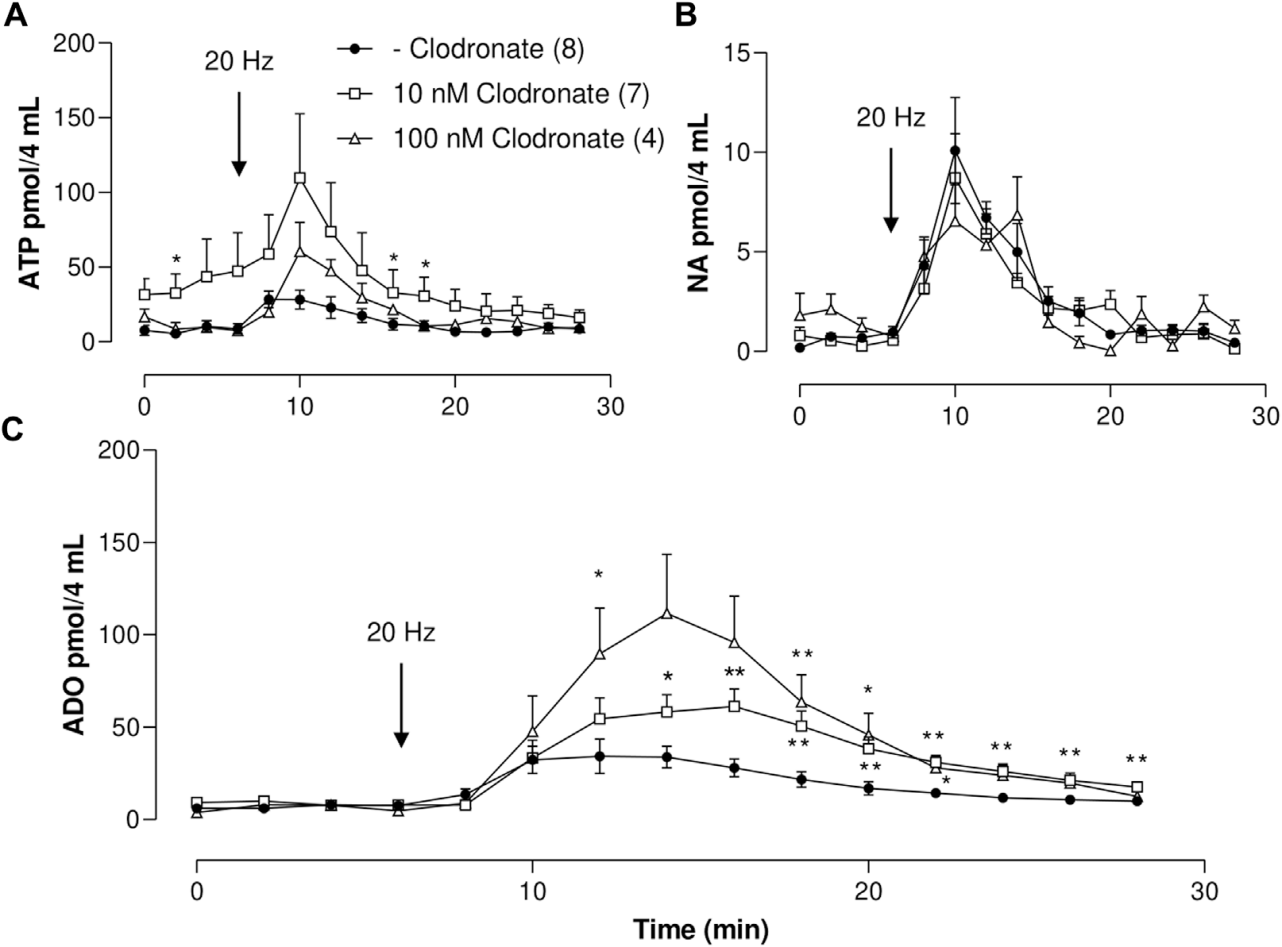
ATP, ADO, and NA outflow to the mesenteric perfusate elicited by electrical nerve endings stimulation in mesenteries perfused in the absence or presence of either 10 or 100 nM clodronate; a time course study. **(A)** Overflow of ATP, **(B)** NA overflow, and **(C)** ADO overflow. Mesenteries were perfused prior to, during, and after nerve endings depolarization, in the absence (closed circles, *n* = 8) or presence of either 10 nM clodronate (*n* = 7, open squares) or 100 nM clodronate (*n* = 4, open triangles). The arrows indicate the initiation of the 1-min nerve endings depolarization (20 Hz, 1 ms trains of 60 V) to elicit co-transmitter release from the nerve terminals. Symbols indicate mean values; bars the S.E.M. *, *p* < .05; **, *p* < .01 unpaired t-test compared to perfusions without clodronate.

Mesentery perivascular nerve endings electrical stimulation elicited an increase in ATP outflow [Figure 1A, one-way ANOVA F (14,105) = 3.723, *p* < .0001]. Notwithstanding, mesentery perfusion with .01–1,000 nM clodronate did not modify the spontaneous overflow nor the total outflow induced by electrical stimulation as compared to perfusions in the absence of bisphosphonate (Table 1). Perfusions with either 10 nM or 100 nM clodronate time responses are shown in Figure 1A, and no ATP outflow reduction induced by clodronate was observed. Values of two-way ANOVA analysis for .01–100 nM clodronate were F(14,165) = 2.004, *p* = .0203; F (14.165) = 1.909, *p* = .0286; F (14.165) = 0, *p* = .1297; F (14.195) = .8745, *p* = .5875, F (14,150) = 1.952, *p* = .0253.

#### ADP

As with ATP, a significant increase in the mesentery perfusate was attained following nerve terminal electrical stimulation [one-way ANOVA F (14,105) = 3.398, *p* = .002]. Mesentery perfusion with .01–1,000 nM clodronate did not modify the spontaneous overflow nor the total outflow induced by electrical stimulation as compared to perfusions in the absence of bisphosphonate (Table 1). These findings are supported by two-way ANOVA for .01–100 nM: F (14.165) = .2800, *p* = .9954; F (14.165) = 1.079, *p* = .3800; F (14.165) = .3450, *p* = .9867 and F (14.195) = 1.032, *p* = .4234; F (14.150) = 1.852, *p* = .0361. We never observed that clodronate reduced purine overflow.

#### AMP

Following perivascular electrical nerve stimulation, we consistently observed an increase in AMP outflow [one-way ANOVA F (14,105) = 2.485, *p* = .0044]. The purine spontaneous outflow was not modified by perfusion with .01–1,000 nM clodronate as compared to those perfused without clodronate (Table 1). Only 100 nM clodronate increases the total overflow elicited by nerve terminal depolarization (Table 1). Values of the two-way ANOVA analysis for .01–1,000 nM clodronate were F (14,165) = .4059, *p* = .9716; F (14,165) = .2444, *p* = .9978; F (14,165) = .7514, *p* = .7199; F (14,195) = .5997, *p* = .8634; F (14.150) = .9479, *p* = .5095; F (14,150) = 1.670, *p* = .0674. These results support the lack of clodronate-induced AMP overflow inhibition.

#### ADO

As with the previous results, perivascular electrical nerve stimulation of mesentery preparations elicited a significant increase in ADO outflow [Figure 1C, one-way ANOVA F (14,105) = 5.958, *p* < .0001]. This ADO increase was delayed as compared to the ATP increase (compare time courses in Figures 1A–C), supporting the notion that the ADO overflow may derive mainly from tissue ectoATPase metabolism. The nucleoside outflow elicited by nerve terminal depolarization did not change following perfusion with .01–1 nM clodronate as compared to those in the absence of clodronate (Table 1). Two-way ANOVA revealed that time courses of ADO outflow for .01–1 nM clodronate values were F (14,165) = .8247, *p* = .6415; F (14,165) = .3784, *p* = .9794; and F (14,165) = .2334, *p* = .9983, indicating no change as compared with tissues perfused in the absence of clodronate. Notwithstanding, only 10 nM clodronate increased ADO outflow (Table 1); the two-way ANOVA value was F (14,195) = 2.693, *p* = .0012. Further increasing clodronate concentration to 100–1,000 nM did not alter the ADO outflow (Table 1).

#### NA

Perivascular electrical nerve stimulation elicited a clear increase in the NA outflow (Figure 1B), the value of one-way ANOVA was F (14,105) = 9.065, *p* < .0001). Moreover, the bioamine outflow was not altered by .01–1,000 nM clodronate as compared to parallel experiments perfused in the absence of bisphosphonate, the results of the two-way ANOVA analysis for .1–1,000 nM clodronate were F (14,165) = .2095, *p* = .9991; F (14,165) = .3042, *p* = .9929; F (14,165) = .6355, *p* = .8325; F (14,195) = .3588, *p* = .9842; F (14,150) = .9465, *p* = .5110, or F (14,150) = .4829, *p* = .939. These values are detailed in Table 1 and support the lack of clodronate-induced inhibition of NA overflow.

### Perfusion with 10 nM clodronate was lengthened to 180 min

Due to the lack of significant clodronate effects on the total overflow of ATP/metabolites or NA elicited by electrical depolarization, we argued that perhaps it was necessary to extend the duration of the perfusion with 10 nM clodronate. In the case of the spontaneous ATP outflow, the value was 1.26 ± .81 pmol (*n* = 4) vs. 2.99 ± 1.04 pmol (*n* = 4), *p* = .239 by the unpaired t-test, following prolonged 10 nM clodronate treatment. The total ATP outflow was increased after nerve depolarization, from 106.73 ± 25.16 pmol (*n* = 4) to 458.82 ± 137.65 pmol (*n* = 4), *p* = .0455 by the unpaired t-test by prolonged 10 nM clodronate perfusion. Neither the spontaneous overflow nor the total ADP, AMP, or NA outflow elicited by transmural perivascular electrical nerve stimulation was modified by clodronate. The spontaneous ADP overflow was from 4.23 ± 2.36 pmol (*n* = 4) to 9.62 ± 1.18 pmol (*n* = 4), *p* = .0873 by the unpaired t-test, while after nerve stimulation, the total ADP values were 300.90 ± 59.27 pmol (*n* = 4) vs. 438.30 ± 125.70 pmol (*n* = 4), *p* = .3513 by the unpaired t-test. Spontaneous AMP overflow values were 2.64 ± 1.34 pmol (*n* = 4) vs. 2.89 ± .82 pmol (*n* = 4), *p* = .8783 by the unpaired t-test. After nerve depolarization, the total AMP control was 363.08 ± 60.52 pmol (*n* = 4), and following 10 nM clodronate treatment, the total AMP was 227.20 ± 33.44 pmol (*n* = 4), *p* = .0970 by the unpaired t-test. In contrast, spontaneous ADO outcome increased 3.9-fold from 1.81 ± 1.19 pmol (*n* = 4) to 7.19 ± 1.63 pmol (*n* = 4), *p* = .0378 by the unpaired t-test; however, the total ADO overflow following electrical nerve depolarization halved from 333.75 ± 40.01 pmol (*n* = 4) to 157.16 ± 48.62 pmol (*n* = 4), *p* = .0310 by the unpaired t-test. Moreover, neither the spontaneous NA outflow (.72 ± .28 pmol, *n* = 4) nor the total evoked by electrical stimulation (32.90 ± 5.35 pmol, *n* = 4) was modified by 10 nM clodronate (.30 ± .21 pmol, *n* = 4), versus 28.66 ± 3.85 pmol, *n* = 4), for the spontaneous outflow and total elicited by electrical stimulation, respectively (unpaired t-test analysis was *p* = .5439 and *p* = .2822, respectively).

### Evans blue reduced the concentration-dependent overflow of ATP/metabolites and NA time course studies

#### ATP outflow

**FIGURE 2:**
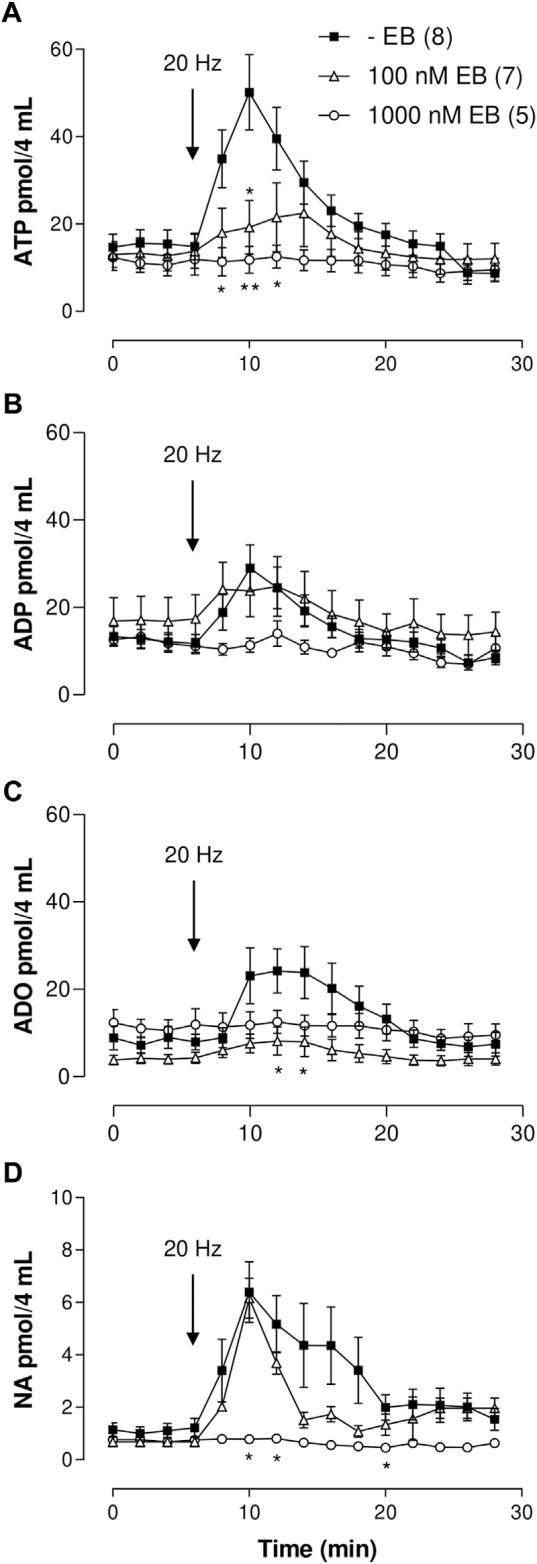
ATP/metabolites or NA mesenteric perfusate outflow time course elicited by sympathetic nerve terminal depolarization in preparations perfused with either 100 or 1,000 nM Evans blue (EB). **(A–D)** show the overflow of ATP, ADP, ADO, and NA, respectively. Tissues were perfused prior to, during, and after nerve depolarization in the absence (closed squares, *n* = 8); or presence of either 100 nM EB (open triangles, *n* = 7) or 1,000 nM EB (open circles, *n* = 5). Arrows indicate perivascular electrical nerve endings stimulation (20 Hz, 1 ms trains of 60 V during 1 min). *, *p* < .05, **, *p* < .01, unpaired t-test as compared to values in the absence of EB.

In sharp contrast with clodronate, mesentery perfusions with 100 or 1,000 nM EB reduced the nucleotide overflow as compared to matched experiments perfused without EB (Figure 2A; Supplementary Table S1). Perivascular electrical nerve endings stimulation of mesenteries elicited an increase in the outflow of ATP [one-way ANOVA F (14,195) = 6.101 *p* < .0001], which was not modified in mesenteries perfused with 1–10 nM EB as compared to those perfused without this dye; two-way ANOVA values for 1 nM or 10 nM EB were F (14,240) = .8986, *p* = .5610 and F (14,255) = 1.220, *p* = .2605, respectively. However, perfusion with 100 or 1,000 nM EB significantly reduced and flattened the ATP overflow elicited by electrical stimulation up to the point that no significance was observed in the nucleotide overflow (one-way ANOVA values for 100 or 1,000 nM EB were F (14,90) = .5647, *p* = .8836 and F (14,60) = .1741, *p* = .9996), respectively, evidencing that 100–1,000 nM EB diminished ATP overflow.

#### ADP outflow

Perivascular nerve terminal stimulation of mesenteries evidenced an increased overflow of this purine [one-way ANOVA F (14,195) = 3.526, *p* < .0001]. As with ATP, perfusion with 1–100 nM EB did not modify the ADP outflow as compared to results with perfusions in EB absence (Supplementary Table S1); two-way ANOVA values for 1, 10, or 100 nM EB were F (14,240) = .7333, *p* = .7398; F (14,255) = .3130, *p* = .9921; and F (14,285) = .3952, *p* = .9757, respectively. Increasing EB to 1,000 nM showed a flattened overflow curve, and an indication of ADP outflow was annulled [one-way ANOVA F (14,60) = .9582, *p* = .5052, Figure 2B; Supplementary Table S1].

#### AMP outflow

Likewise, nerve endings depolarization augmented AMP outflow [one-way ANOVA F (14,195) = 3.585, *p* < .0001]. An amount of 1 nM EB increased the spontaneous and electrically evoked AMP overflow compared to perfusions without EB; the value of two-way ANOVA was F (14,240) = 3.26, *p* < .0001 (Supplementary Table S1). However, mesenteries perfused with 10, 100 or 1,000 nM EB did not show a significant change in the electrically evoked overflow as compared with tissues perfused without EB, as shown in Supplementary Table S1; two-way ANOVA values for 10, 100, or 1,000 nM EB were F (14,255) = .8945, *p* = .5654; F (14,285) = .5306, *p* = .9143; and F (14,255) = .4424, *p* = .9594, respectively.

#### ADO outflow

Electrical nerve terminal depolarization evidenced a delayed but significant ADO overflow increase with respect to ATP overflow [Figure 2C, Kruskal–Wallis test χ^2^ (14) = 28.73, *p* = .0114]. Mesenteries perfused with 1–1,000 nM EB did not show a significant change in the spontaneous overflow as compared with tissues perfused without EB (Supplementary Table S1); 1 nM EB increased the electrically evoked overflow as compared with tissues perfused without EB, and 10–1,000 nM EB reduced the ADO overflow (Supplementary Table S1). Two-way ANOVA values for 1 and 10 nM EB were F (14,240) = .5968, *p* = .8665 and F (14,255) = .4903, *p* = .9373, respectively. Increasing EB to 100 or 1,000 nM flattened the ADO increase; one-way ANOVA values for 100 and 1,000 nM EB were F (14,90) = .7925, *p* = .7396 and F (14,60) = .1741, *p* = .9996, respectively, showing ADO overflow annulment.

#### NA outflow

Electrical stimulation of the perivascular nerve terminals induced a significant increase in NA overflow [Kruskal–Wallis test χ^2^ (14) = 38.04, *p* < .0005]. Perfusion with 1–100 nM EB did not alter the NA output. Two-way ANOVA values for 1–100 nM clodronate as compared to perfusions without EB were F (14,240) = .2259, *p* = .9986; F (14,255) = .6492, *p* = .8221; and F (14,285) = .3740, *p* = .9812, respectively. Increasing EB to 1,000 nM flattened the time course curve [one-way ANOVA F (14,60) = 1.703, *p* = .0789, Figure 2D], indicating complete blockade of the bioamine overflow. Supplementary Table S1 summarizes the results of the spontaneous and electrically evoked overflow.

As a further analysis, when the outflow of ATP or NA elicited by electrical nerve stimulation was compared between the two independent series of protocols performed without clodronate or EB, no statistical differences were observed in the nucleotide outflow in the two-way ANOVA F (14,280) = .8135, *p* = .6543 nor the catecholamine outflow [two-way ANOVA F (14,280) = 1.511, *p* = .1061 for NA], indicating only minor experimental variability between assays occurred.

### DIDS was less potent than EB to reduce ATP/metabolite perfusate overflow

#### ATP outflow

Electrical stimulation of the nerve terminals induced a significant ATP overflow [one-way ANOVA F (14,135) = 3.890, *p* < .0001]. DIDS proved less potent than EB to reduce the output of electrically evoked ATP output since while 1 µM DIDS perfusion did not modify its output as compared to tissues perfused without DIDS [two-way ANOVA F (14,210) = .8138, *p* = .6537]; however, 10 µM DIDS flattened the nucleotide output [one-way ANOVA F (14,75) = .1907, *p* = .9993], highlighting a substantial reduction in the nucleotide outflow induced by electrical stimulation (Supplementary Table S1).

#### ADP outflow

Likewise, electrical stimulation of the nerve terminals induced a significant ADP overflow [one-way ANOVA F (14,135) = 2.128, *p* = .0138]. Perfusion with 1–10 µM DIDS did not modify the ADP overflow compared with tissues perfused without DIDS [two-way ANOVA F (14,210) = .2722, *p* = .9961; F (14,210) = 1.665, *p* = .0649], as shown in Supplementary Table S1.

#### AMP outflow

Nerve endings electrical stimulation induced a significant AMP increase in its overflow [one-way ANOVA F (14,135) = 2.682, *p* = .0017]. However, neither perfusion with 1 nor 10 µM DIDS modified the AMP overflow induced by electrical nerve depolarization as compared to the tissues perfused without DIDS. Two-way ANOVA analysis values for 1 or 10 µM DIDS were [F (14,210) = .7700, *p* = .7009 and F (14,210) = .1262, *p* = .9999], respectively, as shown in Supplementary Table S1.

#### ADO outflow

Electrical stimulation of the nerve endings increased ADO overflow [Kruskal–Wallis test χ^2^ (14) = 27.36, *p* = .0173]. An amount of 1 µM DIDS shifts the outflow curve up, and the two-way ANOVA value is [F (14,210) = 1.980, *p* = .0203]. However, 10 μM DIDS did not change the nucleoside outflow [F (14,210) = .6313, *p* = .8380] as compared to the perfusions performed in the absence of DIDS. Supplementary Table S1 summarizes the results of the spontaneous and electrically evoked overflow.

#### NA outflow

Electrical stimulation of the nerve endings induced a significant NA overflow [Kruskal–Wallis test χ^2^ (14) = 37.68, *p* = .0006]. However, 1 or 10 µM DIDS showed a tendency toward a reduction in the catecholamine outflow, and the values did not reach statistical significance as compared with tissues perfused without DIDS (Supplementary Table S1). The two-way ANOVA values for 1 µM or 10 µM DIDS were F (14,210) = .3745, *p* = .9728; F (14,210) = .6468, *p* = .8235, respectively.

### Effect of reserpine treatment on spontaneous and electrically evoked ATP/metabolites and NA outflow

Pretreatment with .2 mg/kg reserpine did not modify the spontaneous nor the electrically evoked overflow of ATP/metabolites or NA as compared to the vehicle (Supplementary Table S2). However, 2 mg/kg reserpine which did not modify the spontaneous outflow of ATP/metabolites or NA decreased by 60% the total perfusate ATP overflow or 84% the NA outflow elicited by nerve endings depolarization (Supplementary Table S2), without modifying ADP, AMP, or ADO. The 2 mg/kg reserpine pretreatment flattened the increase in ATP overflow induced by nerve terminal stimulation [one-way ANOVA F (14,60) = 1.681, *p* = .0840]; likewise, it obliterated the NA overflow as evidenced by the one-way ANOVA [F (14,60) = 1.082, *p* = .3920], supporting the ATP and NA overflow annulment.

### Changes in perfusion pressure elicited by electrical depolarization of perivascular mesenteric nerve endings in tissues perfused with clodronate, EB, and DIDS or the perfused mesenteries of reserpine-treated rats

**FIGURE 3:**
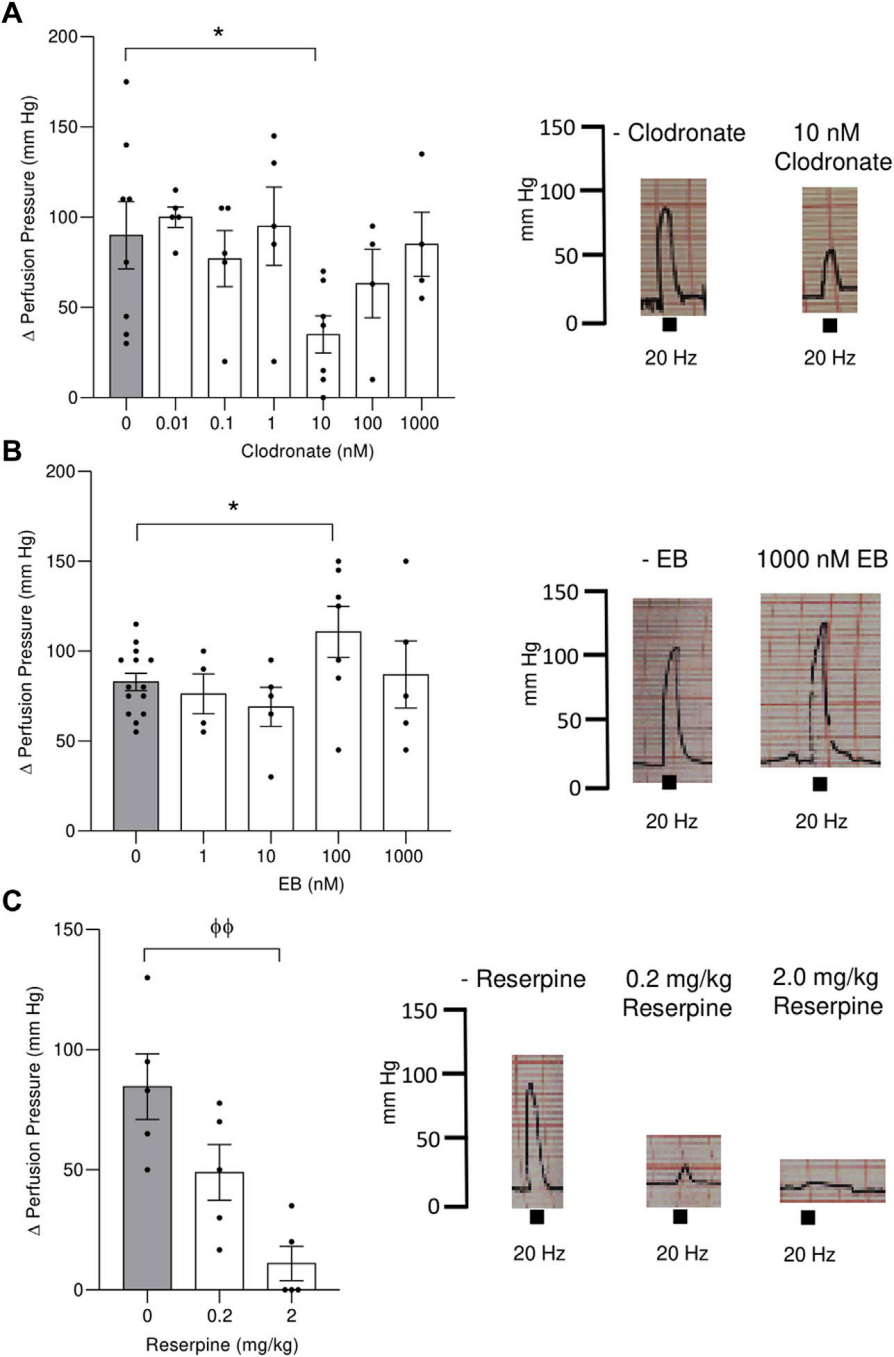
Electrical stimulation evoked an increase in the perfusion pressure of the mesenteric bed vascular territory and its modification following perfusion with either clodronate or Evans blue (EB) or mesenteries from rats pretreated with reserpine for 48 h. Left panel show columns depicting the increase in the mesentery perfusion pressure elicited by electrical nerve endings stimulation (20 Hz train of electrical pulses) expressed as the Δ increase in the mesentery perfusion pressure in preparations perfused with several clodronate concentrations, upper **(A)**; Evans blue middle **(B)** or rats pretreated with .2 or 2 mg/kg reserpine for 48 h lower **(C)**. Right panel shows representative polygraphic traces of the changes in perfusion pressure recordings, with the respective calibrations (mm Hg). Columns represent mean values; bars the S.E.M. Gray columns represent matched tissues without drugs. Each closed circle in the columns represents experimental values obtained from separate rats. * *p* < .05, unpaired t-test; Φ Φ, *p* < .01, Mann–Whitney test, as compared to tissues without drugs.

Electrical nerve depolarization was associated with an abrupt increase in the mesentery perfusion pressure. This increase ensued within the first seconds of electrical depolarization, returning to baseline few seconds after stopping the 1-min nerve terminal stimulation. Representative vasomotor tracings are shown in Figure 3.

1) Clodronate: The increase in perfusion pressure elicited by nerve terminal electrical stimulation in preparation perfused without this drug was 90.00 ± 18.61 mm Hg (*n* = 8), a value that was reduced to 35.00 ± 10.35 mm Hg (*n* = 7, *p* = .028, unpaired t-test) in preparations perfused with 10 nM clodronate (Figure 3A). Neither smaller nor larger clodronate perfusate concentrations modified the increase in perfusion pressure evoked by nerve terminal stimulation (Figure 3A). Parallel experiments which prolonged the 10 nM clodronate perfusion for 180 min did not modify the increase in perfusion pressure elicited by nerve depolarization (83.33 ± 21.2, *n* = 4) vs. perfusions without clodronate (95.00 ± 5.40 mm Hg, *n* = 4), unpaired t-test *p* = .6135.
2) EB: Although perfusions with 1,000 nM markedly reduced the overflow of ATP/metabolites and NA, it did not modify the increase in perfusion pressure elicited by nerve terminal stimulation. However, perfusion with 100 nM showed a minor but significant increase in the vasomotor response (Figure 3B) compared to tissues perfused without this dye. We infer that despite the reduced outflow of ATP/metabolites and NA, the co-transmitter concentration that reaches the neuroeffector junction causes the full vasomotor response.
3) DIDS: Perfusions with 1 or 10 μM did not change significantly the motor responses elicited by electrical nerve endings stimulation. The Δ perfusion pressure (mm Hg) in tissues without the drug was 110.6 ± 11.98 (*n* = 10), while the increase in perfusion pressure averaged 100.29 ± 28.28 (*n* = 6) in tissues perfused with 1 μM DIDS or 73.26 ± 18.97 (*n* = 6) following 10 μM DIDS.
4) Reserpine: A gradual dose-dependent decrease in the increase of the perfusion pressure elicited by nerve endings depolarization was observed in mesenteries of animals pre-treated with either .2 or 2 mg/kg reserpine, compared to the respective vehicles (Figure 3C). While the .2 mg/kg reserpine pretreatment only attenuated the elicited increase in perfusion pressure, a 10-fold higher dose caused a consistent and profound reduction of the vasomotor responses from 84.60 ± 13.70 (*n* = 5) to 11.00 ± 7.14 mm Hg (*n* = 5), Mann–Whitney test, *p* = .0079.

### Extracellular ATP/metabolites in primary cultures of endothelial cells incubated with .01–1,000 nM clodronate

To assess and confirm whether clodronate increases ADO in isolated endothelial cells, primary cultures of the rat mesentery endothelium were prepared to assess clodronate effects in non-neuronal mesentery components. Endothelial cell incubation with .01–100 nM clodronate increased the spontaneous extracellular ADO, while 300 or 1,000 nM clodronate did not. Moreover, only 1,000 nM clodronate augmented the spontaneously released extracellular ATP and ADP. These results are summarized in Table 2. Moreover, following mechanical stimulation, a procedure used to elicit purine release to the cell media release, 10 nM clodronate significantly increased extracellular ATP, ADP, AMP, and ADO (Table 2). Increasing the bisphosphonate to 100 nM or 1,000 nM only modified extracellular ADO (Table 2). As with the perfusion studies, 10 nM clodronate or higher augmented extracellular ADO. No clear clodronate concentration dependence was observed for ADO, except that perhaps clodronate elicited a bell-shaped curve.

**TABLE 2:** Clodronate concentration-dependent effects on the spontaneous and mechanically evoked ATP/metabolites from primary cultures of endothelial cells from the rat mesentery.

	Spontaneous extracellular purines expressed as % of purines in the absence of clodronate; X ± S.E.M.							
Clodronate nM								
	0 (n=28)	0.01 (n=6)	0.1 (n=8)	1 (n=8)	10 (n=12)	100 (n=10)	300 (n=10)	1000 (n=15)
ATP	93.37 ± 8.58	45.12 ± 9.19*	75.08 ± 13.23	91.87 ± 11.87	91.48 ± 10.51	94.33 ± 18.13	100.40 ± 31.21	165.00 ± 28.67**
ADP	100.00 ± 7.94	108.28 ± 27.25	144.34 ± 18.11*	129.84 ± 18.73	113.38 ± 9.80	206.05 ± 57.53 **	106.52 ± 33.60	205.56 ± 26.59****
AMP	100.00 ± 7.12	87.94 ± 13.93	92.16 ± 7.8	80.32 ± 17.14	94.08 ± 6.78	109.26 ± 25.81	114.59 ± 22.31	88.04 ± 12.76
ADO	100.00 ± 7.04	150.27 ± 16.53*	144.21 ± 17.78*	156.55 ± 23.79**	137.14 ± 7.78**	190.54 ± 44.14**	155.31 ± 31.79	131.57 ± 15.95

Mechanical stimulation-induced ATP/metabolite release, expressed as % purines in the absence of clodronate; X ± S.E.M.								
Clodronate nM								
	0 (n=28)	0.01 (n=6)	0.1 (n=8)	1 (n=8)	10 (n=9)	100 (n=9)	300 (n=12)	1000 (n=14)
ATP	98.46 ± 9.24	106.30 ± 22.02	107.58 ± 27.67	126.86 ± 21.00	136.44 ± 10.29*	127.61 ± 26.59	94.41 ± 13.09	98.12 ± 11.47
ADP	100.00 ± 7.36	87.87 ± 9.81	85.14 ± 8.00	103.52 ± 11.83	141.89 ± 9.87**	144.27 ± 27.14*	101.62 ± 10.43	108.11 ± 6.54
AMP	100.00 ± 6.82	90.59 ± 16.08	80.18 ± 5.10	111.31 ± 12.95	154.92 ± 15.67***	117.73 ± 22.12	91.07 ± 12.69	83.37 ± 8.83
ADO	100.00 ± 3.74	73.42 ± 8.28**	116.32 ± 33.94	102.94 ± 15.07	122.46 ± 9.71 Φ	177.33 ± 34.09***	105.00 ± 8.98	134.60 ± 10.94ΦΦ

Numbers in parenthesis indicate the cell wells analyzed from five separate rats. *, *p* < .05; **, *p* < .01; ***, *p* < .001; ****, *p* < .0001; unpaired t-test as compared to cells without clodronate. Φ, *p* < .05; ΦΦ, *p* < .01, Mann–Whitney test as compared to cells incubated without clodronate addition.

### eATP hydrolysis time course protocols confirm lack of clodronate-induced inhibition of ATP metabolism and of eADO metabolism

To examine whether clodronate (1–1,000 nM) modified endothelial cell extracellular ATP metabolism, eATP time course studies were followed, and eATP hydrolysis and metabolic by-products were determined in parallel. Parallel protocols examined whether eADO is enzymatically degraded or transported intracellularly by isolated endothelial cell cultures.

**FIGURE 4:**
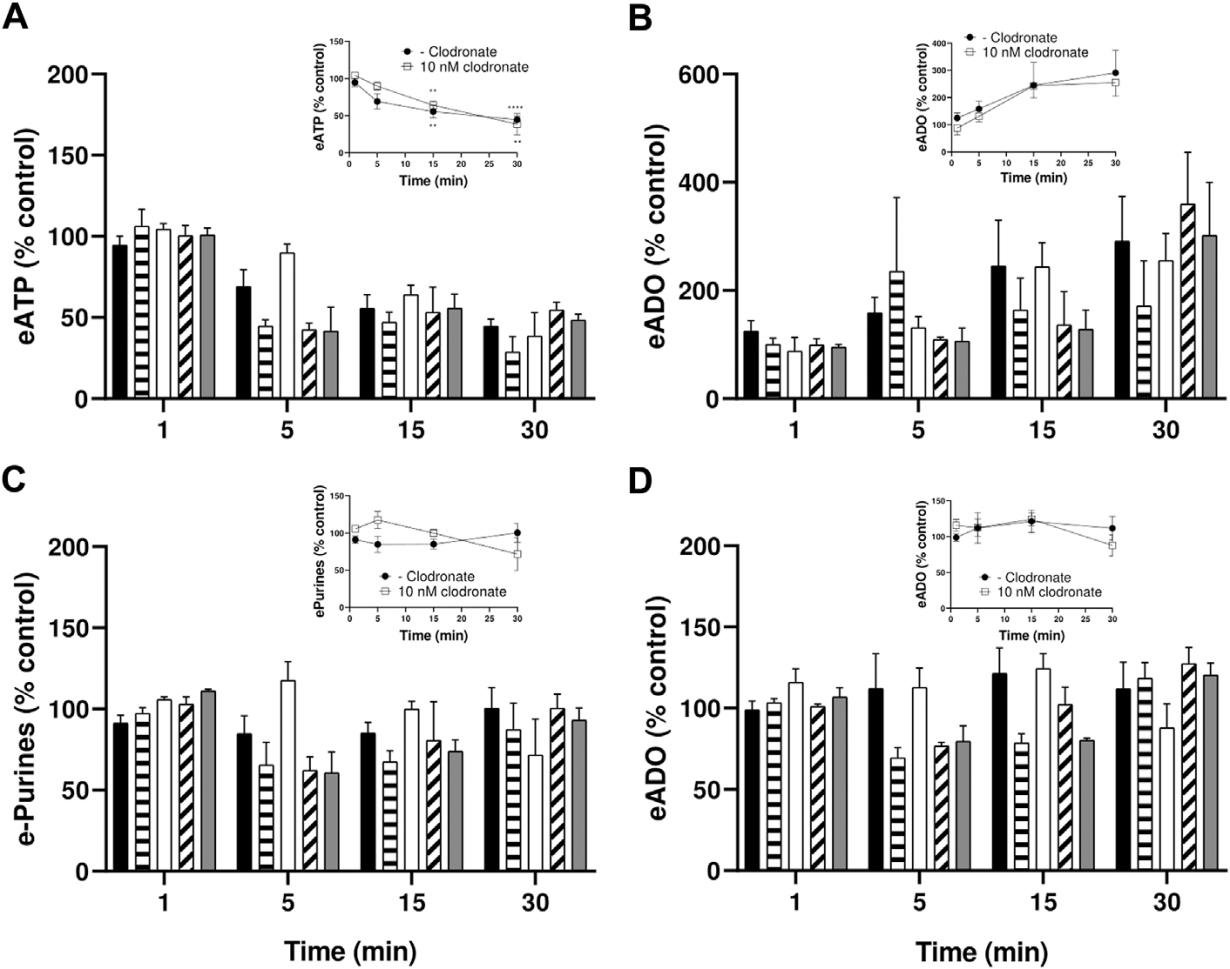
Endothelial cells metabolize eATP but not eADO, lack of inhibition by 1–1,000 nM clodronate. To ascertain whether clodronate interferes with ectoATPase cell metabolism, cultures of mesentery cells were incubated with eATP, and its metabolism was followed over 30 min. **(A)**Decay of 50 mM eATP added to the extracellular media. **(B)** shows the corresponding eADO accumulation in cells treated with eATP, while **(C)** depicts the total etheno purines from the same cells depicted, evidencing that the extracellular etheno purines were maintained over time. In contrast, **(D)** shows the application of 25 nM eADO to a separate, but parallel, set of endothelial mesentery cells. Closed columns represent controls in the absence of clodronate (*n* = 8), dashed columns depict 1 nM clodronate (*n* = 4), open columns 10 nM clodronate (*n* = 6), oblique dashed columns 100 nM clodronate (*n* = 4), and gray columns 1,000 nM clodronate (*n* = 4). Bars in the columns show the S.E.M. No significant difference was observed when comparing controls versus the clodronate effect at each incubation time. Insets of these Figures show the effect of 10 nM clodronate (open squares, *n* = 6) compared to a parallel set of cells incubated in the absence of the bisphosphonate (closed circles, controls, *n* = 8). Symbols indicate the mean S.E.M. **, *p* < .01, and ****, *p* < .0001 unpaired t-test compared to the corresponding 1-min controls.

eATP is a substrate of ectoATPases; therefore, it is metabolized extracellularly by cell ectoenzymes with a profile similar to ATP. eATP decayed with a half-life of approximately 15 min, generating as by-products eADP, eAMP, and eADO (Figure 4A); moreover, 1–1,000 nM clodronate did not interfere with the eATP metabolic degradation nor affected its half-life (Figure 4A). The inset of Figure 4A depicts the effect of 10 nM clodronate and its statistical analysis compared to the first minute of eATP addition. During the 30-min protocol, eADO increased 2–3 fold (Figure 4B, inset shows 10 nM clodronate effect). Total extracellular etheno purines, (the addition of eATP plus eADP, eAMP, and eADO) did not change over time (Figure 4C), suggesting no significant intracellular etheno derivative transport.

In contrast to eATP, eADO is not an ADA substrate nor apparently transported intracellularly, explaining that extracellular eADO remained intact during the 30-min incubation protocol (Figure 4D). In addition, 1–1,000 nM clodronate did not significantly modify eADO in the extracellular media (Figure 4D).

### Endothelial cell ADA activity

**FIGURE 5:**
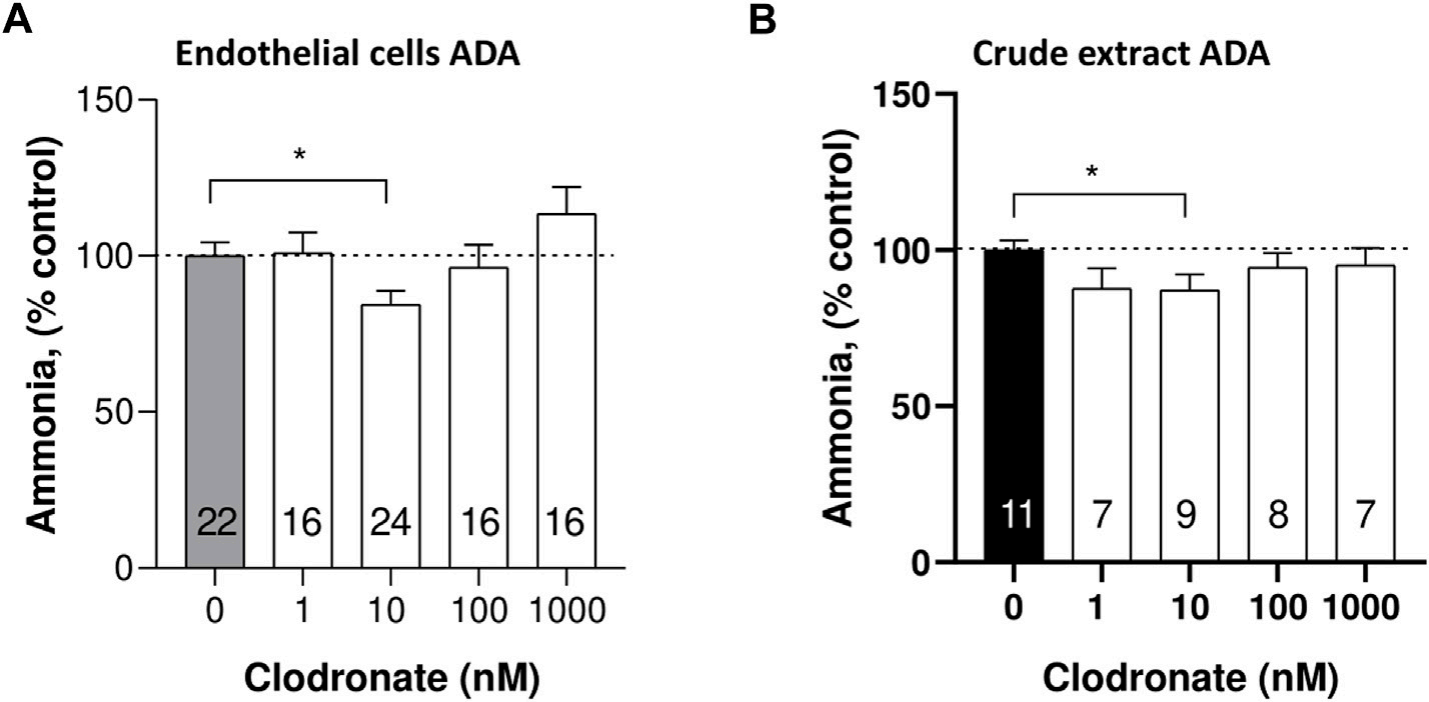
Comparison of ADA activity inhibition by 10 nM clodronate in mesentery endothelial cells or a crude calf intestinal enzyme preparation. To assess whether clodronate interferes with ADA activity, mesentery cells **(A)** or a crude calf intestine extract **(B)** was used to examine putative enzyme inhibition elicited by clodronate. ADA activity was determined *via* a colorimetric titration of the ammonia generated as a byproduct of enzyme reaction. Columns indicate the mean values and bars the S.E.M. of multiple enzyme assays performed in the absence or presence of 1–1,000 nM clodronate. Numbers inside the columns refer to the separate mesentery cell wells assays or crude enzyme activity assay. Results are expressed as % ammonia formed compared to parallel controls without clodronate. *, *p* < .05 unpaired t-test, compared with control, without clodronate.

To directly evaluate whether clodronate interferes with the enzymatic ADO conversion to inosine, a subset of 24-well plate endothelial cells was incubated with exogenous ADO as a substrate in the absence or presence of varying clodronate concentrations. Clodronate failed to modify ADA activity, except for 10 nM, a concentration that slightly, but consistently reduced enzyme activity (15.5 ± 4.3%, unpaired t-test, *p* = .0128, Figure 5A).

As positive controls, the cells were incubated with either 10 or 30 μM quercetin, which elicited a 5 ± 6.0 (unpaired t-test, *p* = .6833) and 41.8 ± 2.4% (unpaired t-test, *p* < .0001) reduction of ADA activity, respectively.

### Crude extract ADA activity

To ascertain more directly the effect of clodronate on the enzyme activity, we used a commercial crude enzyme extract and examined whether clodronate reduced the activity of ADA. In this enzyme preparation, 10 nM clodronate caused a modest but significant 12.8 ± 4.8% (*p* = .049, Figure 5B) inhibition of the enzyme activity a result consistent with the observation of clodronate-induced inhibition of cell enzyme protocols (Figure 5A). Enzyme activity was examined using .3 mM exogenous ADO and 6.25 mU/mL ADA. Two positive controls were used in this protocol, one was .7 mM adenine, which inhibited 28.6 ± 6.2% (*n* = 9, *p =* .001); likewise, 1 mM quercetin inhibited ADA activity 13.9 ± 4.7% (*n* = 9, *p* = .0283), results compatible with the 10 nM clodronate-induced inhibition.

## Discussion

Perfusion of the rat mesentery with clodronate did not reduce the outflow of ATP/metabolites and NA induced by electrical stimulation of perivascular sympathetic nerve endings from the rat mesentery vascular bed, an unexpected result considering clodronate’s alleged VNUT inhibitor profile. On the contrary, a modest increase in purine overflow was shown. Notwithstanding, in matched parallel control studies, mesentery bed perfusion with EB or DIDS evidenced a significant reduction in the ATP outflow, raising doubts that clodronate is a VNUT inhibitor in sympathetic nerve endings. EB is a symmetric azo dye, currently recognized as the most potent VNUT inhibitor available (Moriyama et al., 2017); DIDS also inhibited VNUT but with markedly less potency than EB, agreeing with current findings. We are aware that neither EB nor DIDS are VNUT selective since a variety of non-VNUT effects have been consistently described, including VGLUT inhibition documented by Hartinger and Jahn, (1993), Roseth et al. (1995) and revised by Eriksen et al. (2016). In addition, EB has long been used for clinical purposes such as the estimation of human blood volume, vascular permeability, and lymph node detection as recently reviewed by Yao et al. (2018), revealing multiple targets. The precise mechanism of how EB or DIDS elicit VNUT inhibition remains unclear. Sawada et al. (2008) observed that both EB and DIDS inhibited concentration-dependently the ATP uptake activity and also reported that the transporter is chloride-dependent. In addition, Zalk and Shoshan-Barmatz, (2006) reported that the ATP uptake observed in isolated brain synaptic vesicles was DIDS-sensitive; hence, whether this effect is due to VNUT activity remains to be determined.

We are aware that the use of a perfused vascular bed to detect the outflow of ATP and co-transmitters from the perivascular sympathetic nerve endings has limitations. Notwithstanding, this preparation was used extensively by autonomic investigators to examine the neurochemistry of the adrenergic and purinergic synapses and to quantify the outflow of these transmitters (Donoso et al., 2006; Donoso et al., 2018b). The present results do not allow the characterization of clodronate, EB, or DIDS as VNUT inhibitors, even though both EB and DIDS reduced concentration-dependently the outflow of ATP and NA from the mesentery bed, a profile expected of VNUT inhibitors. Only clodronate increased ADO outflow, suggesting a separate target, which we deem could be related to ADA inhibition. EB concentrations used in the current experiments are within reported VGLUT IC_50_ values (Roseth et al., 1995; Thompson et al., 2005) or the purified VNUT SLC17A9 assay in the synthetic *in vitro* liposomes of the Sawada et al. (2008) study. Higher DIDS concentrations were required to inhibit VNUT in the liposome reconstituted bioassay or VGLUT (Hartinger and Jahn 1993), a finding substantiated by current data. Moreover, in cultured mesentery-derived endothelial cells, Donoso et al. (2018a) reported that EB decreased the spontaneous extracellular cell medium ATP. This latter effect is apparently due to inhibition of vesicular ATP release mediated by an EB-sensitive mechanism, an action not observed in similar protocols using clodronate.

To account for the consistent clodronate-evoked ADO accumulation observed in the perfusion studies, we examined whether clodronate interferes directly or indirectly, with purine metabolism or transport both in the mesenteric bed or isolated endothelial cells and the crude calf intestine ADA preparation. ADO accumulation must result from either lack of its metabolism or cellular transport by these cells. Following this reasoning, we next examined whether clodronate induced inhibition of ADA activity in endothelial cells, a finding that may explain the increase in ADO following clodronate mesentery perfusion or in isolated endothelial cells. A modest yet reproducible and significant reduction in ADA activity was consistently observed only with 10 nM clodronate. The mechanism of inhibition was not further addressed, except we confirmed that plant polyphenols such as quercetin inhibited ADA activity at micromolar concentrations, in agreement with Melzig, (1996). Interestingly, only 10 nM clodronate, but not other concentrations, consistently inhibited the enzyme closely mimicking the observation in mesentery endothelial cells. Although we do not understand why the inhibition is exclusively observed with 10 nM clodronate, three separate observations support our contention that 10 nM clodronate elicits this effect: 1. in the perfused mesentery preparation, 10 nM clodronate elicited an ADO increase paralleled by a decreased neuroeffector motor response induced by transmural nerve depolarization, 2. this concentration increases extracellular ADO in endothelial cells upon mechanical stimulation, and 3. 10 nM clodronate inhibits ADA activity in a commercial crude enzyme preparation. Moreover, in the Moriyama assay, 15 nM clodronate is the half median concentration required to inhibit VNUT activity.

In addition, current findings are consistent with previous findings that both quercetin and adenine inhibit ADA activity, apparently by a competitive mechanism (Melzing, 1996; and Alunni et al., 2008), which supports and highlights the clodronate findings. We are certain that clodronate does not interfere with ATP or eATP metabolism, except it modestly inhibits ADO degradation to inosine. The present results do not allow discarding that clodronate could eventually reduce ADO transport, but eADO results do not support this proposal. It should be emphasized that eADO is neither an ADA substrate nor an ADA inhibitor (Jamal et al., 1988); therefore, since eADO remains constant in the enzyme incubation protocol, we infer that eADO is not an ADO transporter substrate (Figure 4D). To directly support this hypothesis, we are open to further investigations. Notwithstanding, we infer that the bisphosphonate-induced ADO increase in the perfused mesentery is of functional relevance since 10 nM clodronate elicited a reduction in the increase in perfusion pressure evoked by electrical stimulation of sympathetic nerve endings. Curiously, larger clodronate concentrations did not follow this inhibitory pattern. Increasing clodronate to 100 nM not only increased ADO but also ADP and AMP, which may hinder the ADO-induced vasodilatation since ADP may constrict the mesentery vessels, causing a physiological antagonism of the ADO-evoked vasorelaxation. In this vascular bed, endogenous ADO has a strong vasodilator efficacy. Neither EB nor DIDS modified the increase in perfusion pressure elicited by electrical nerve endings depolarization, even though both chemicals reduced electrically evoked co-transmitter outflow. ATP is known to elicit a mixed dual contractile and relaxant effects related to P2X and P2Y receptor activation (Ralevic and Burnstock, 1998). Furthermore, endothelial cell P2Y receptor activation induces nitric oxide production (Donoso et al., 2018b), which may add to the ADO-induced vasodilatation. Since we are fully aware that the 40-min clodronate mesentery perfusion might not be enough to elicit a significant VNUT inhibition, the duration of the 10 nM clodronate perfusion was extended for 180 min. Notwithstanding, we did not observe a reduction in nucleotide overflow nor a further decrement in the increase of the perfusion pressure induced by electrical stimulation. On the contrary, the ADO outcome was reduced, suggesting that the increase in ADO overflow, elicited by nerve terminal depolarization, occurred within the 40-min clodronate mesentery perfusion. In contrast to 10 nM clodronate, EB which decreased the outflow of ATP and NA did not reduce the increase in mesentery perfusion pressure elicited by perivascular electrical nerve stimulation. This observation allows us to deduce that the response is clodronate-specific and that the vasomotor effect elicited by perivascular nerve stimulation must be caused by a small fraction of the released transmitters since it was not observed after 1,000 nM EB. We are conscious that transmitter outflow is the result of compounded and balanced effects between transmitter release, degradation, uptake, and transmitter diffusion through the smooth muscle and endothelium to finally reach the vascular lumen, where ATP/metabolites and NA were determined. Although EB markedly reduced the release of sympathetic co-transmitters, the fraction that reaches the neuroeffector junction must suffice to cause a full vasomotor response elicited by perivascular nerve terminal stimulation.

To further investigate why the EB-induced reduction in the outflow of ATP/metabolites and NA was not paralleled by a reduction of the mesentery perfusion pressure, additional experiments were conducted in reserpinized rats. Reserpine elicited a concentration-dependent reduction in the increase in perfusion pressure associated with a marked decrease in ATP and NA outflow to levels comparable to those elicited by 1,000 nM EB. We are aware that the effect of reserpine was examined after 2 days of treatment (Huidobro-Toro, 1985), where adaptive mechanisms may occur such as a reduction in other sympathetic co-transmitters involved in the vasomotor response. Reserpine is used as a NA vesicular transporter marker (VMAT), in analogy to EB, a VNUT marker. This concept may help identify whether the same synaptic vesicle stores both ATP and NA or whether separate vesicle pools store independently sympathetic co-transmitters. This is an ongoing debate for several decades without a firm final demonstration. An exciting immunohistochemical study using selective color labeled antibodies for the vesicular NA transporter and VNUT recently concluded that in the rat tail artery, the NA and ATP transporter storage vesicles may be distinct and associated each with voltage-dependent calcium channel subtypes (Mojard Kalkhoran et al., 2019). These findings highlight the notion of apparently separate storage vesicles for each co-transmitter. Notwithstanding, ATP is required for NA storage (Estevez-Herrera et al., 2016), a result at variance with this structural contention, an issue that needs further research. Moreover, extracellular ATP regulates chromaffin vesicular content (Larsson et al., 2019), a finding that augments the complexity of co-transmitter storage, a fact that is not disputed for a single transmitter. Independent of the synaptic vesicle’s dynamics in sympathetic nerve endings, selective vesicular transporter inhibitors such as reserpine for VMAT, or EB for VNUT, are valuable neuroscience tools for further clarifying multi-transmitter vesicular storage in sympathetic nerve terminals. An exciting venue that supports the physiological role of VNUT in purinergic transmission derives from transgenic VNUT knockout mice (VNUT^−/−^). Transgenic mice are phenotypically apparently normal (Sakamoto et al., 2014), but several experimental lines using these animals demonstrate that VNUT-mediated vesicular ATP release is key for the storage and release of ATP. Moreover, gene silencing results in purinergic transmission impairment (Moriyama et al., 2017; Miras-Portugal et al., 2019; Tatsushima et al., 2021). The relevance of VNUT in chronic neuropathic pain, glaucoma, and other sympathetic nerve system-related diseases poses the opportunity of targeting drugs to ATP transporters, as a novel therapeutic goal of future medical applications.

Is it plausible that the mechanism of the analgesic effect of clodronate in chronic pain may be related to the observed ADO accumulation? This observation may be of clinical relevance since clodronate has a chronic pain analgesic profile (Kato et al., 2017) and ADO elicits analgesia (Sollevi, 1997; Jung et al., 2022). As to whether our finding of ADO accumulation, likely due to nanomolar clodronate-induced reduction in the ADA activity, is somehow involved in the analgesic effect of clodronate remains to be better examined.

Clodronate is a pyrophosphate analog of biomedical relevance used for over 60 years in the treatment of bone-related diseases. Clodronate, due to its two phosphates, likely chelates calcium, with a low permeability coefficient. In Caco-2 cells, the clodronate transport value was .25 × 10^–7^ cm/s (Raiman et al., 2001), a non-favorable physicochemical variable to freely cross cell membranes, favoring our view that clodronate likely interacts with an extracellular site of action, unless it is mobilized intracellularly by phosphate transporters. In chondrocytes, a cell model used to study articular cartilage degeneration and improve arthritis management, clodronate is claimed to enter cells by pinocytosis, rather than simply diffusing or using a transporter (Rosa et al., 2014). An observation we have not fully resolved is the lack of consistent concentration-dependence of the clodronate effects. This issue likely indicates that clodronate has complex pharmacokinetics and may target several mechanisms simultaneously, indicative of compounded pharmacodynamics as well.

In summary, present findings highlight a minor, though consistent ADA inhibition which might account for the observed ADO accumulation as well as the reduction in the vasomotor effect elicited by nerve endings stimulation observed with 10 nM clodronate. The role of clodronate as a nanomolar ADA inhibitor may play a relevant role *in vivo* as an ADO modulator. Moreover, the present results do not allow characterizing clodronate as a VNUT inhibitor. Drugs which target vesicular transporters are valuable neuroscience tools to solve the dynamics of transmitter storage and recycling plus the compounded complexities of multiple co-transmitter storage mechanisms and release in sympathetic co-transmission, an exciting venue for future research.

## Data Availability

The original contributions presented in the study are included in the article/Supplementary Material; further inquiries can be directed to the corresponding author.
