# Supplementary material for: Nanomolar clodronate induces adenosine accumulation in the perfused rat mesenteric bed and mesentery-derived endothelial cells
Source: Front Pharmacol. 2023 Jan 20;13:1031223. doi: 10.3389/fphar.2022.1031223 (PMC9895365; doi:10.3389/fphar.2022.1031223)
Supplement: Supplementary file 1 [file Table1.pdf]

Supplementary Table 1

Evans blue (EB) and DIDS concentration-dependent effects on spontaneous and electrically evoked ATP/metabolites and NA outflow from the mesentery neuroeffector junction

Spontaneous overflow (pmol), X ± S.E.M.

	Without drugs	EB				DIDS	
	(n=18)	1 nM (n=4)	10 nM (n=5)	100 nM (n=7)	1000 nM (n=5)	1 µM (n=6)	10 µM (n=6)
ATP	15.25±2.49	12.78±2.04	20.55±4.67	13.20±3.41	11.48±2.74	12.88±3.74	15.93±4.90
ADP	14.60±2.03	19.48±3.78	26.04±6.35*	17.04±5.46	12.21±1.76	29.00±13.73	25.68±12.00
AMP	16.46±2.52	30.88±6.38Φ	25.93±6.43	6.93±2.09Φ	13.04±4.62	23.43±6.51	28.79±9.15
ADO	8.98±1.68	15.85±3.33	7.23±1.31	4.07±1.17	11.48±2.74	17.13±5.97	12.07±3.75
NA	0.91±0.16	1.69±0.22*	2.35±0.58	0.68±0.17	0.21 ±0.08ΦΦ	2.06±0.17**	1.26±0.12

Total overflow elicited by electrical nerve depolarization (pmol), X ± S.E.M

	Without drugs	EB				DIDS	
	(n=18)	1 nM (n=4)	10 nM (n=5)	100 nM (n=7)	1000 nM (n=5)	1 µM (n=6)	10 µM (n=6)
ATP	99.49±20.55	124.42±2.75	37.66±13.52	37.89±15.87Φ	5.48±1.49ΦΦΦ	59.64±26.29	25.56±9.21Φ
ADP	43.55±10.07	83.22±32.39	22.38±6.19	43.74±15.42	11.79±4.83 Φ	49.94±24.08	49.48±11.18
AMP	33.46±6.17	85.92±8.11**	27.06±8.59	53.33±26.90	57.37±29.29	45.88±25.89	73.60±23.93
ADO	56.88±13.82	130.32±30.37Φ	16.18±5.52Φ	22.51±12.41Φ	5.48±1.49ΦΦΦ	32.71±17.19	28.52±14.22
NA	20.91±4.32	12.52±5.50	5.29±1.20Φ	17.39±2.44	2.65 ± 0.32 ΦΦ	10.70±3.47	13.76±6.45

In parenthesis, number of preparations assessed. *, p<0.05; ** p<0.01, Unpaired t test as compared to tissues perfused without drugs. Φ, p <0.05, ΦΦ, p< 0.01, ΦΦΦ, p <0.001, Mann Whitney test as compared to tissues perfused without drugs.
