# Supplementary material for: Nanomolar clodronate induces adenosine accumulation in the perfused rat mesenteric bed and mesentery-derived endothelial cells
Source: Front Pharmacol. 2023 Jan 20;13:1031223. doi: 10.3389/fphar.2022.1031223 (PMC9895365; doi:10.3389/fphar.2022.1031223)
Supplement: Supplementary file 2 [file Table2.pdf]

Supplementary Table 2

Effects of reserpine pretreatment on spontaneous and electrically evoked ATP/metabolites and NA overflow from the mesentery neuroeffector junction

X ± S.E.M. (pmol)

	Spontaneous outflow				Total outflow elicited by electrical nerve depolarization		
	Vehicle	Reserpine pre-treatment			Vehicle	Reserpine pre-treatment	
	(n=5)	0.2 mg/kg (n=3)	2 mg/kg (n=5)		(n=5)	0.2 mg/kg (n=3)	2 mg/kg (n=5)
ATP	8.45 ±1.66	23.33 ±10.48	15.86 ±3.10		86.12 ±6.28	63.49 ±36.55	40.76±10.49*
ADP	10.65 ±1.48	22.57 ±6.48	9.98 ±1.55		32.03 ±6.82	28.17 ±8.80	38.17 ±8.29
AMP	14.19 ±2.57	14.28 ±4.91	12.86 ±4.25		52.27 ±9.47	20.05 ±3.45	36.67 ±11.95
ADO	4.66 ±1.53	10.78 ± 7.79	6.58 ±1.38		55.23 ±16.77	39.49 ±20.02	40.83 ±8.73
NA	1.41 ±0.62	1.35 ± 0.13	2.53 ± 0.71		26.68 ±8.59	16.47 ± 5.73	4.27 ± 1.16*

In parenthesis, number of preparations assessed. *, $p < 0.05$, unpaired t test as compared to the vehicle pretreatment.
